# Assessment of Durability and Degradation Resistance of Geopolymer Composites in Water Environments

**DOI:** 10.3390/ma18163892

**Published:** 2025-08-20

**Authors:** Kacper Oliwa, Barbara Kozub, Katarzyna Łoś, Piotr Łoś, Kinga Korniejenko

**Affiliations:** 1 Faculty of Material Engineering and Physics, Cracow University of Technology, Jana Pawła II 37, 31-864 Cracow, Poland; kacper.oliwa@student.pk.edu.pl (K.O.); barbara.kozub@pk.edu.pl (B.K.); 2 Department of Machine Parts and Mechanism, Faculty of Mechanical Engineering, Technical University of Liberec, Studentská 1402/2, 46117 Liberec, Czech Republic; katerina.los@tul.cz (K.Ł.); piotr.los@tul.cz (P.Ł.); 3 Polytechnic Faculty, University of Kalisz, Nowy Swiat Str. 4, 62-800 Kalisz, Poland

**Keywords:** geopolymer, amphibolite, water environment, underwater application

## Abstract

This article presents experimental studies on the characterization of geopolymer composites intended for applications in aquatic environments, with particular emphasis on underwater infrastructure. The motivation for conducting the research was the growing need to develop durable and ecological building materials that will be resistant to long-term exposure to moisture and aggressive chemical agents, typical for the underwater environment, where traditional cement concretes undergo gradual degradation due to long-term water impact, including hydrotechnical and underwater infrastructure. Geopolymer binders were produced based on metakaolin activated by alkaline solutions containing sodium hydroxide. Several series of mixtures with additives such as blast furnace slag, amphibolite and carbon fibers were developed to evaluate the effect of these components on mechanical strength, water absorption and chemical durability. The conducted studies showed that slag additions improved mechanical properties, for the best composition it across 50 MPa. In contrast, the addition of amphibolite had an unfavorable effect, which probably results from introducing inhomogeneity into the material structure. The presence of carbon fibers promoted matrix cohesion, but their uneven distribution could lead to local strength differences. Water absorption tests have shown that geopolymers reach full water saturation within 24 to 48 h, which indicates rapid establishment of capillary equilibrium and limited further water penetration. The conclusions from the work indicate that geopolymer composites with a moderate amount of blast furnace slag and subjected to appropriate curing conditions. High strength, water and chemical resistance make them suitable for, among others, the construction of marine foundations, protection and structural shields of submerged applications.

## 1. Introduction

In the face of growing pressure for sustainable development and the need to reduce carbon dioxide emissions, alternatives to traditional construction materials are being explored. Concrete is widely recognized as a fundamental building material. However, its extensive use has a significant environmental impact due to the high carbon emissions associated with cement production. Global cement production amounts to approximately 4 billion tons annually, leading to nearly the same amount of CO_2_ emissions into the atmosphere [1]. It is worth noting that cement production is responsible for about 7% of total anthropogenic CO_2_ emissions worldwide. It is estimated that the annual global production of concrete is around 10 billion cubic meters, making it the primary material used for infrastructure development across the globe [2]. Due to the projected growth of the global population, an increased demand for essential infrastructure such as housing, transportation systems, and public facilities is also anticipated. The rising needs in these areas will likely lead to a further increase in concrete production, which in turn will intensify CO_2_ emissions and exacerbate the environmental issues associated with climate change. In recent years, geopolymers have gained significant attention as environmentally friendly construction materials due to their low carbon footprint, high mechanical strength, and excellent durability. Compared to traditional Portland cement [3], the production of geopolymers can significantly reduce CO_2_ emissions while also allowing for the utilization of large amounts of industrial solid waste, offering both environmental and economic benefits [4,5].

Specific extreme marine environments place very high demands on the corrosion resistance of construction materials. Key challenges that require urgent solutions in the context of geopolymer applications include ensuring high strength and resistance to brittle fracture in marine environments, as well as optimizing their durability through modification of the material structure [6]. Traditional geopolymers based on fly ash and slag require highly alkaline activation systems, which, in the presence of reactive aggregates, may promote alkali–aggregate reactions in marine environments and also cause degradation of the passive layer on reinforcing steel [7,8].

During curing, geopolymers exhibit susceptibility to shrinkage caused by moisture evaporation and chemical reactions, which promotes the formation of microcracks, expansion, and structural cracking [9]. Under cyclic wetting and drying conditions, especially in the tidal zone, this can lead to delamination at the material–steel interface, reducing structural integrity and long-term durability [10,11].

A review of the literature shows that previous studies on the durability of alkali-activated materials, including geopolymers, have demonstrated their considerable resistance to corrosive agents such as acids, sulfates, chlorides, and carbon dioxide [12,13]. Geopolymers exhibit greater resistance to acid corrosion than Portland cements, especially those with low calcium content [14,15]. Due to their low permeability and the characteristic structure of the C–A–S–H gel, it is possible to slow down acid penetration and maintain structural continuity [12,16]. Nevertheless, the processes of dealumination and gel structure transformation require further investigation, particularly in the context of using different types of slag as alkali-activated precursors. Sulfate corrosion behaves differently in alkali-activated binders compared to Portland cements, primarily due to the distinct chemistry of their binding phases [13]. Geopolymers often show higher resistance to sodium sulfate attack; however, their susceptibility to magnesium sulfate—especially in the presence of calcium—leads to a loss of mechanical properties. Reactions in low-calcium materials and the impact of internal sulfate corrosion, a phenomenon particularly relevant to hazardous waste immobilization, also remain not fully understood. In the case of chloride penetration, pore structure and pore solution chemistry play a key role [17]. The results can be misleading, as they are influenced more by the ionic conductivity of the pore solution than by actual permeability. Differences stemming from the type of activator used and the nature of the gel significantly affect chloride diffusion. Carbonation in alkali-activated materials also differs from that in Portland cements [18,19]. In low-calcium systems, reactions mainly occur in the pore solution without significant changes to the N–A–S–H gel, whereas in high-calcium systems, structural disintegration of the C–A–S–H gel may occur [20]. Accelerated carbonation tests do not always yield realistic results, as overly aggressive conditions may lead to an overestimation of the actual carbonation rate. Moreover, the mechanisms of strength loss due to carbonation, as well as the influence of humidity and sample age on the development of this process, are still insufficiently understood [20,21].

Although alkali-activated materials offer many durability advantages, many of their degradation mechanisms are not yet fully understood [22,23]. The complexity of their chemical composition, the influence of different activators, changes in pore structure, and interactions with ions present in various environments highlight the need for continued research into their long-term durability and behavior under real operating conditions [24,25]. Developing appropriate testing methods and gaining a proper understanding of degradation mechanisms are essential for the effective large-scale implementation of these materials in the construction industry. Despite the previous research, there is still a research gap in the area of investigations on long-term chemical resistance and microstructural stability of alternative binders under aquatic exposure.

The aim of this study is to design new geopolymer compositions with improved longevity and environmental performance and to determine their resistance to degradation in aquatic environments. The study analyzes seven geopolymer-based compositions in terms of water absorption and compressive strength. Moreover, the microstructure investigations, including FTIR and SEM allow for an in-depth analysis of the mechanisms occurring in the materials. The conducted work aimed to assess the potential use of geopolymer materials for infrastructure applications in inland water environments, including polluted reservoirs, as well as in marine conditions. To investigate the impact of different environments on geopolymers, water absorption tests were conducted in various solutions: distilled water, sodium chloride solution, hydrochloric acid solution, a mixture of acids, and sodium hydroxide solution. Compressive strength tests of the samples were performed before and after exposure to different aquatic environments. To examine the morphology of the samples and changes in their chemical structure before and after water absorption, SEM, and FTIR analyses were used.

## 2. Materials and Methods

### 2.1. Sample Preparation

Seven different geopolymer mixtures were prepared for the study at the Technical University of Liberec. The base component of the composites was metakaolin. In all samples, the activator used was 0.9 kg of sodium water glass, and 0.01 kg of carbon fibers was also added. The granulometric analysis was performed for basic materials. The particle size distribution of metakaolin exhibited a D50 of 9.458 µm, with a broad distribution ranging from D10 of 1.9055 µm to D90 of 23.853 µm. The relatively fine particle size (mean size 11.895 µm) contributed to a higher surface area, potentially enhancing the geopolymerization reaction [26]. The silica dioxide powder had a narrow particle size distribution, with a D50 of 9.969 µm, D10 of 4.483 µm, D90 of 17.942 µm, and a mean value of 11.131 µm. The cellulose has relatively large particles, with a D50 of 125.381 µm, D10 of 47.090 µm, D90 of 226.467 µm, and a mean value of 139.374 µm.

The samples were divided into two series. The first series consisted of four samples to which silicon dioxide (SiO_2_) and slag in amounts ranging from 0.3 kg to 1 kg were added. The second series, consisting of three samples, did not contain silicon dioxide. One sample included only the basic materials, while the other two contained, respectively, 1 kg of slag and 1 kg of amphibolite. The composition of the samples is presented in Table 1.

All the samples were prepared according to the same scheme:

- Firstly, the metakaolin was mixed with the activator (sodium water glass) and mixed for 5 min.
- In the next step, the short carbon fibers were added to the mixture. The process was continued for 3 min.
- After that time, the proper amount of silicon dioxide was included in the paste. The mixing process was continued for 3 min (for the composition without silica, this step was omitted).
- Then, the mixture was continued for 2 min with the addition of cellulose (for the composition without cellulose, this step was omitted).
- In the last step, the amphibolite and slag were added to the paste. The mixing process was continued next 3 min. After that time, the process was ended.

The samples were cast and cured at an ambient temperature for 28 days before testing.

### 2.2. Methods

Density was determined as a ratio of mass to volume— the geometric method. Volume was calculated based on the arithmetic means of the measurements for 15 cuboidal samples.

To evaluate the granulometric properties of the raw materials, a comprehensive particle size distribution analysis was performed using a laser diffraction technique on the Anton Paar PSA 1190 LD Graz particle analyzer (Anton Paar GmbH, Graz, Austria).

The chemical composition (elemental and oxide) of the supplied samples was determined using X-ray fluorescence (XRF) spectroscopy. The analysis was carried out with a PUMA S2 spectrometer (Bruker, Billerica, Massachusetts, USA). An appropriate amount of powder was placed in a plastic holder on a 4 μm-thick polypropylene film. Measurements were performed in an air atmosphere.

To examine the water absorption capacity of the geopolymers, a water absorption test was conducted. The purpose of this test is to determine how much water the material can absorb. This allows for an assessment of its resistance to the harmful effects of water, such as expansion during freezing or chemical degradation [27]. The samples were immersed in five different solutions:

- Distilled water;
- Sodium chloride solution (992 g H_2_O + 8 g NaCl);
- Hydrochloric acid solution (997.26 g H_2_O + 2.74 g HCl 38%);
- Acid mixture (994.47 g H_2_O + 1.54 g HNO 36.5% + 1.25 g CH_3_COOH 80% + 2.74 g HCl 38%);
- Sodium hydroxide solution (990 g H_2_O) + 10 g NaOH).

The dry samples were fully immersed in water for 30 days. The mass of each sample was measured daily for the first 11 days, until mass stabilization was observed, and then again on days 20, 21, and 30. pH measurements of the solutions on days 10 and 20 revealed a shift to strongly alkaline levels (13–14) in all samples. To simulate real conditions, the solutions were replaced with fresh ones after the pH measurements. Subsequently, water absorption as a function of time was determined.

To assess the strength of the reference and post-absorption samples, a compressive strength test was carried out using a C077N testing machine (Matest, Treviolo, Italy) designed for concrete testing. Cube-shaped samples with dimensions of 30 × 30 × 30 mm^3^ were prepared for the test. The test was performed on 3 samples from each series, and the average of the obtained results was calculated.

The samples were placed in the central part between the plates. The maximum force required to cause failure (F) was recorded, and compressive strength was calculated based on the following equation:

(1) $$\sigma =\frac{F}{A},$$

where

σ—compressive strength [MPa]

F—maximum force [N]

A—cross-sectional area [mm^2^]

Fourier Transform Infrared Spectroscopy (FTIR) is an analytical method used to identify functional groups and the composition of molecular mixtures. The test was performed using a Thermo SCIENTIFIC IS5 FTIR spectrometer with a diamond ATR ID7 attachment (Nicolet iS5, Thermo Scientific, Loughborough, UK).

Prior to testing, the samples were ground using a ZM 200 ultra-centrifugal mill (Retsch GmbH, Haan, Germany). To carry out the measurement, the ATR plate was first cleaned with acetone to remove any contaminants. This step was repeated before each measurement. The powdered samples were then placed on the ATR plate, in its central area where the diamond is located.

The microstructure of the samples was analyzed using a scanning electron microscope (SEM). To make the geopolymer surfaces electrically conductive, a thin layer of gold was applied. The analysis was carried out using a JSM-IT200 scanning electron microscope (JEOL, Tokyo, Japan). mA DII-29010SCTR Smart Coater (JEOL, Tokyo, Japan) was used for gold coating of the samples.

## 3. Results

### 3.1. Density

The results for density measurements are presented in Table 2. The measurements were made for 25 samples. The average value is presented.

### 3.2. Chemical Composition

Based on the XRF analysis, the elemental and oxide compositions were determined. The results for elemental analysis are presented in Table A1 (Appendix A.1) and results for oxide composition are presented in Table 3.

The results obtained in elemental and oxide analysis are coherent. It can be observed that the main elements present in all samples are silicon, aluminum, and calcium. The highest aluminum content was found in sample GEO V5, indicating the absence of this element in the slag and amphibolite additives. Samples GST1 and GEO V7 have the highest silicon content, at 41.83% and 42.59%, respectively. Sample GEO V5 shows the highest potassium content, while samples GST1–7 demonstrate that potassium levels decrease with increasing slag content. These components are typical of geopolymer structure and the calcium component allows for the curing process in ambient temperature [28,29]. The iron content reveals that GEO V5 has the lowest amount of iron at 3%, while all other samples contain more, with GEO V6 and GST6 having the highest amounts at 9.78% and 8.10%, respectively. The calcium content is similar across all samples.

The results of the oxide analysis are in line with elemental analysis. Silicon dioxide is dominant in all samples, reaching the highest values in GST 1 at 55.19% and GEO V7 at 56.95%. The high content of silicon dioxide indicates a strongly siliceous character of all geopolymers. Based on the literature, it can be pointed out that optimal SiO_2_ to Al_2_O_3_ molar ratios around 3 to 3.8 and appropriate calcium level content produce maximum strength and densification of the geopolymer network [30,31]. Based on the chemical composition of raw materials, the geopolymer mixtures were designed to avoid the shrinkage or cracking of the material caused by a large amount of slag or silica dioxide [30,31].

Similarly to the elemental composition, iron oxide content is higher in samples containing slag, GST1 at 8.82%, GEO V6 at 6.70%, and in metakaolin-based GEO V7 at 3.04% compared to the sample without additives, GEO V5, which has only 1.81%.The composition is useful for the geopolymerization process [32,33].

### 3.3. Absorption Tests

Based on the absorption measurements, the values presented in Table 4 were obtained. It presents a comparison of the sample masses before immersion in the solutions and the final mass measurements (average for 3 samples). Additionally, the percentage values of water absorption were calculated.

Based on the analysis of the obtained results, it is evident that sample GEO V5 exhibits the lowest susceptibility to absorption of solution media. The average absorption value for this sample is 2.9%, indicating that the material is highly resistant to deep penetration. For several other samples, the water absorption values were around 6%, suggesting high stability of the material in varying solution environments. Comparing the results for the sample GEO V5 and sample GEO V6, we may notice that the difference in composition is a slag that was added to the composition of GEO V6. The results suggest that the slag significantly increased the water absorption in the sample as well as decreased the limitation for corrosive environments such as alkali, acids, and salts. However, some authors noticed a decrease in water absorption as a result of slag addition [34], others research confirm that increasing the water absorption increases caused by the slag addition [35]. It should be stressed that the behavior of the materials can be influenced by a particular type of slag used and its treatment, especially important feature in this case is the tendency of the materials with slag addition to higher porosity [36].

The sample series labeled GST 5 showed the highest value of this characteristic parameter, exceeding 10%, which suggests the presence of mechanisms in this material that promote increased penetration. A likely explanation for this phenomenon is the presence of blast furnace slag, which is known for its high porosity, making it a good adsorbent. Additional components that increase the water absorption can be cellulose and silicon dioxide. It is visible when we compare the results for the sample GEO V6 and GST 1, where the difference in composition is between these two components. The higher water absorption, as well as the absorption of other solutions, is visible for sample GST1. The influence of cellulose and silicon dioxide is not such strong as in the case of slag, but the difference is statistically important. In this case, the influence of cellulose, which has its hydrophilic nature, is rather clear—it increased the water absorption [37]. In the case of silicon dioxide, data from the literature are not clear. The literature confirms that silicon dioxide should seal the composite structure, but it could also have an influence on the Si/Al ratio and the influence of the curing process. In the case of too large an amount of silica, the geopolymerization reaction may not be fully effective, and then the material properties decrease [38].

**Figure A1:**
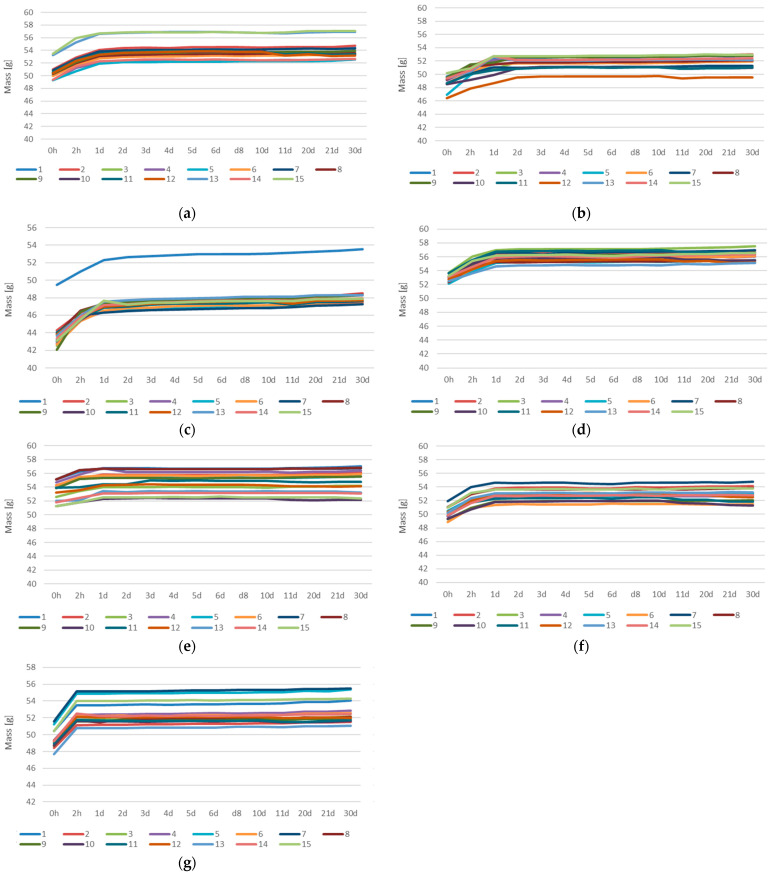
Water absorption over time: (**a**) GST1; (**b**) GST3; (**c**) GST5; (**d**) GST7; (**e**) GEOV5; (**f**) GEOV6; (**g**) GEOV7.

In Appendix A.1 (Figure A1), the water absorption over time for all measured samples is shown. The graphs indicate a general trend of rapid initial surface absorption during the first few hours of immersion. This is followed by a gradual stabilization of the material, with mass increases becoming slower and more consistent over time. For most samples, a significant level of saturation was reached within the first day, after which the absorption process continued at a much slower rate.

### 3.4. Compressive Strength

**Figure 1:**
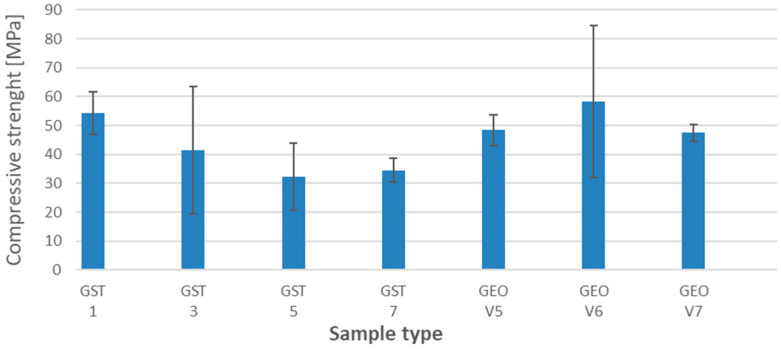
The results of compressive strength for reference samples after 28 days.

Figure 1 presents the results of the compressive strength tests for the samples cured in the laboratory (not submerged). The comparison of values for different samples was a basic point (reference sample) for the determination of compressive strength after degradation in different environments.

**Figure 2:**
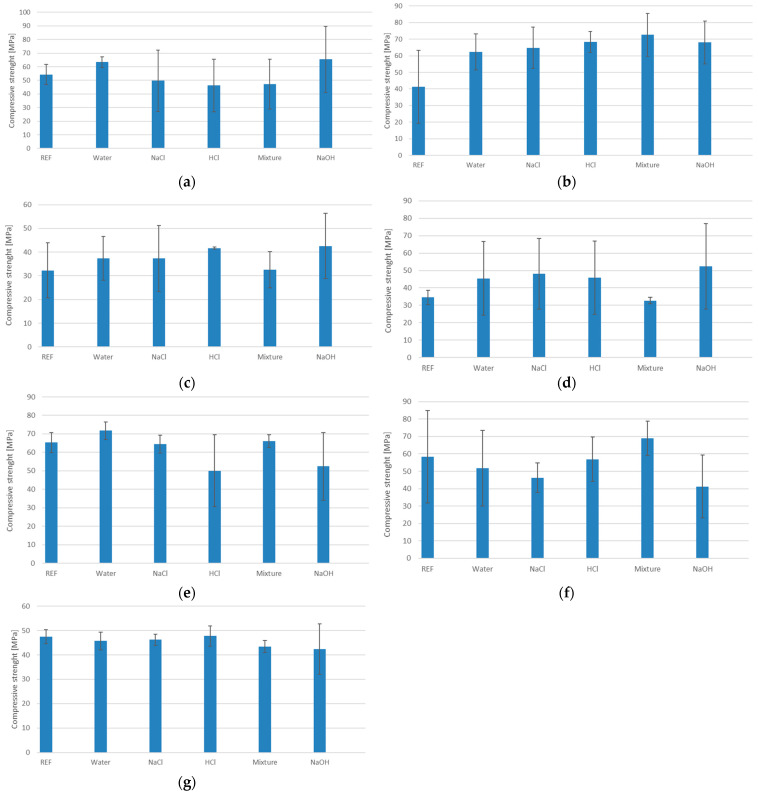
The results of the compressive strength test after water absorption test: (**a**) GST1; (**b**) GST3; (**c**) GST5; (**d**) GST7; (**e**) GEOV5; (**f**) GEOV6; (**g**) GEOV7.

Figure 2 shows the average values obtained from a minimum of 3 samples per series, including error bars representing the measurement uncertainties for all sample series for the samples from different series after degradation in different environments.

Based on the analysis of the test results presented in Figure 2, significant differences in compressive strength are observed depending on the type of solution in which the samples were immersed.

The highest compressive strength values were observed for series GST 1 and GEO V6, which contained the highest amount of slag in their composition. The mechanical resistance of these materials remained high after exposure to various solutions, indicating a high stability of the geopolymer matrix and strong bonding of the reactive phase. Additionally, the small measurement errors suggest a high uniformity of the material.

Samples with reduced slag content, i.e., GST 3, GST 5, and GST 7, exhibited a gradual decrease in compressive strength. The greatest sensitivity, manifested by a drop in strength, was observed in the acidic environment.

In sample GEO V6, which shares the same base composition as GEO V5 but is enriched with slag, better results were observed. The compressive strength was higher, and the average value remained stable despite exposure to aggressive media. This case supports the observation that slag content plays a crucial role in determining the durability of the geopolymer structure.

The analysis of sample GEO V7, which was the only one containing amphibolite, revealed lower compressive strength compared to the GEO V6 series. A noticeable decrease was observed in the acidic environment. This observation provides grounds for the conclusion that amphibolite not only weakens the structural stability of the geopolymer material, but may also act as a destabilizing factor that negatively affects its chemical resistance. These results highlight that both the material composition and the environmental conditions to which the material is exposed have a significant impact on the mechanical properties of geopolymer materials. Particular attention should be given to controlling the amount and inclusion of blast furnace slag, as it contributes both to chemical resistance in acidic and alkaline environments and to increased compressive strength. Amphibolite, present in only one of the series, brought no mechanical benefit. Additionally, the low carbon fiber content and their agglomeration may have led to inconsistent results, as reflected by the large standard deviations observed in the compressive strength charts.

### 3.5. FTIR Analysis

**Figure 3:**
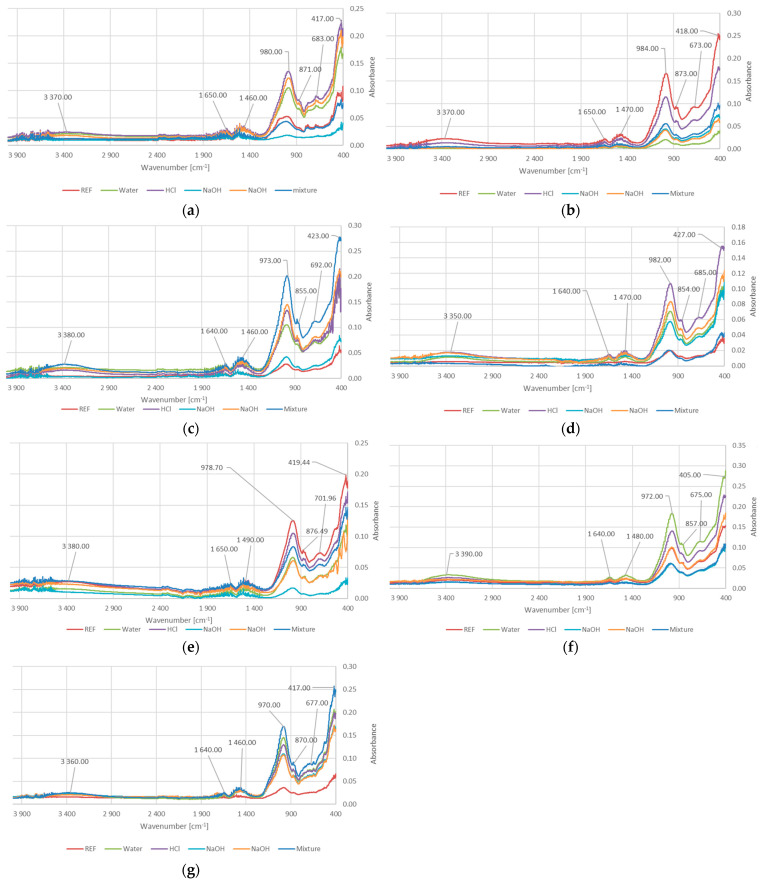
The results of FTIR analysis for: (**a**) GST1; (**b**) GST3; (**c**) GST5; (**d**) GST7; (**e**) GEOV5; (**f**) GEOV6; (**g**) GEOV7.

Figure 3 presents the FTIR spectra of the reference samples as well as the samples after the water absorption tests in various solutions.

As shown in Figure 3, the bands observed at the wavenumber range of 3350–3390 cm^−1^ correspond to the stretching vibrations of free O–H groups. Similarly, the bands at 1640–1670 cm^−1^ are attributed to the bending vibrations of hydrogen-bonded O–H groups [39]. This type of bonding may be influenced partly by the inherent moisture present in geopolymer materials and partly by interactions between surface hydroxyl groups of the geopolymer and water molecules absorbed during testing. It is especially visible for FTIR for GEOV7, when the peaks for reference samples (dry) are significantly lower than for others.

The range of 972–984 cm^−1^ is associated with the asymmetric stretching vibrations of Si–O–T bonds (where T = Si or Al) [40,41]. The 854–876 cm^−1^ bands are related to the bending vibrations of Si–O–Al bonds [40,41]. These kinds of bonds are important for geopolymerization process, because of incorporation of aluminum into the silicate chains results in the formation proper material structure. In this process, the presence of Ca^2+^ also plays an important role—it stabilizes these bonds (balancing charges in silicate network) and helps promote cross-linking within the gel formation (calcium-aluminosilicate hydrate—C-A-S-H phases). Thanks to this, the cross-linking leads to a denser, more cohesive network structure [42,43]. This gives an effect of denser material with better mechanical properties, including compressive strength (Figure 1).

The bands within 405–427 cm^−1^ and 675–702 cm^−1^ correspond to the bending and symmetric stretching vibrations of Si–O–Si bonds, respectively [44]. These bonds are crucial, taking into consideration the mechanism of the geopolymerization process. The geopolymerization reaction involving silicic acid (Si(OH)4), which plays a crucial role in the formation of Si-O-Si bonds, especially these bonds, is responsible for the formation of the structure of the geopolymer network [45,46]. The FTIR peaks observed at approximately 405–427 cm^−1^ are generally attributed to Si-O-Si bending vibrations, especially corresponding to bending modes where oxygen atoms flex within the silicon-oxygen framework. The peaks between 675 and 702 cm^−1^ are probably reflecting angular deformation modes associated with the aluminosilicate network [45,46]. These peaks show the formation and strengthening of the geopolymer network and are also influenced by mechanical properties [45,46]. The formation of Si-O-Si bonds can also influence slag and silicon dioxide content. The combination of slag and high silica content strengthens the geopolymer matrix by reducing pore volume and filling microstructural voids, which enhances the density and consistency of the geopolymer network [31,47]. The results are visible in compressive strength results (Figure 1), the best values were obtained for samples with a high content of slag (GEOV6 and GST1).

The opposite phenomenon can occur when amphibolite is added (GEOV7). It weakens the Si-O-Si bonds and negatively affects on coherence of the network in geopolymers. systems primarily by interfering with the geopolymerization process and disrupting the formation of an aluminosilicate network, especially because this mineral contains a significant amount of crystalline phases that are less reactive or even inert in alkaline activation conditions [32]. This reduces the compressive strength of gopolymers with amphibolite; however, this phenomenon has not beenfully investigated yet.

The 1480–1490 cm^−1^ bands indicate the presence of carbonates, specifically the asymmetric stretching vibrations of C–O bonds.

All samples exhibit a similar set of characteristic vibrational bands, indicating the presence of functional groups typical of aluminosilicate-based materials. The peak around 1480 cm^−1^ in the alkaline samples suggests possible carbonation, leading to the formation of Ca_2_CO_3_.

### 3.6. Microstructural Analysis

**Figure 4:**
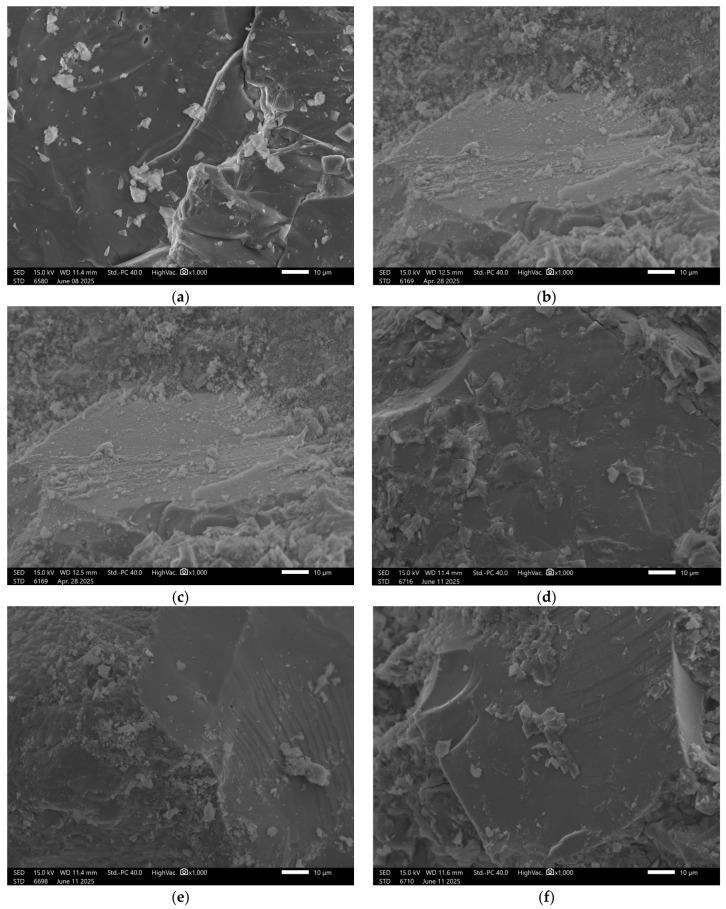
SEM Image for composition GST1: (**a**) reference sample; (**b**) H_2_O; (**c**) H_2_O + NaCl; (**d**) H_2_O + HCl; (**e**) H_2_O + HCl + CH_3_COOH + HNO_3_; (**f**) H_2_O + NaOH.

Recorded images obtained using scanning electron microscopy (SEM) enabled analysis of both reference samples and those subjected to water absorption in the specified solutions. The study focused on visually assessing the influence of the chemical environment on the surface structure and porosity of the geopolymer materials. The surface of the reference sample GST 1 shows a compact and uniform structure, Figure 4.

Numerous fine, evenly distributed grains of the reactive phase were observed, indicating the occurrence of alkaline activation reactions with ground granulated blast furnace slag (GGBFS). Samples exposed to distilled water showed no visible changes—the surface remained intact. In contrast, exposure to the NaCl solution resulted in localized cracking and small surface defects, suggesting the initiation of degradation. More advanced damage was observed after exposure to hydrochloric acid (HCl) and the acid mixture. In these cases, the structure exhibited extensive erosion, with visible cracks and deterioration along phase boundaries, indicating insufficient resistance of the material in low-pH environments. In the sodium hydroxide (NaOH) solution, SEM images revealed surface corrosion typical for geopolymers, manifested by numerous secondary pores and irregular surface texture.

**Figure 5:**
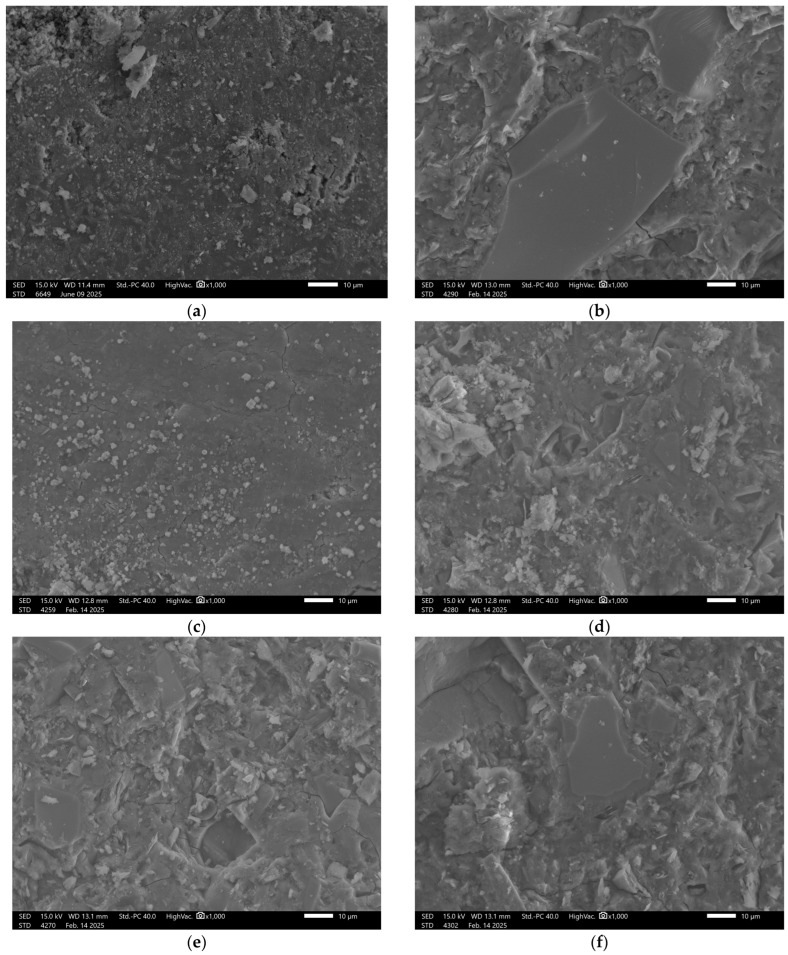
SEM Image for composition GST3: (**a**) reference sample; (**b**) H_2_O; (**c**) H_2_O + NaCl; (**d**) H_2_O + HCl; (**e**) H_2_O + HCl + CH_3_COOH + HNO_3_; (**f**) H_2_O + NaOH.

In the case of the GST 3 sample, which contained a lower amount of slag, the reference material’s surface exhibited a higher number of pores and a lower presence of consolidated phases, Figure 5.

**Figure 6:**
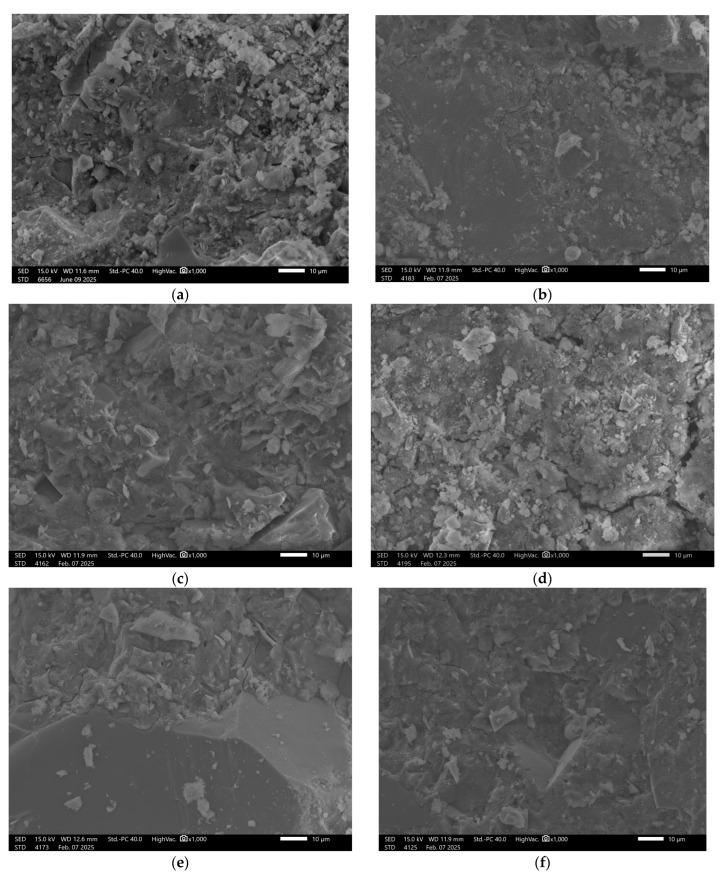
SEM Image for composition GST5: (**a**) reference sample; (**b**) H_2_O; (**c**) H_2_O + NaCl; (**d**) H_2_O + HCl; (**e**) H_2_O + HCl + CH_3_COOH + HNO_3_; (**f**) H_2_O + NaOH.

Upon exposure to distilled water and sodium chloride (NaCl), interfacial cracking was observed in addition to visible surface porosity. In acidic and alkaline environments, the recorded surface underwent significant degradation. This was manifested by pronounced leaching and fragmentation of the matrix, indicating reduced chemical resistance of the material under extreme pH conditions. The beneficial effect of increased ground granulated blast furnace slag (GGBFS) content is confirmed in the images of sample GST 5 (Figure 6), where improved structural cohesion is clearly visible. The surface of the control sample exhibited fewer pores compared to GST 3. Samples exposed to various solutions showed similar types of defects as those observed in GST 3—acidic and alkaline environments led to noticeable structural breakdown. In contrast, exposure to distilled water resulted in only minor surface alterations, indicating relatively stable performance under neutral conditions.

**Figure 7:**
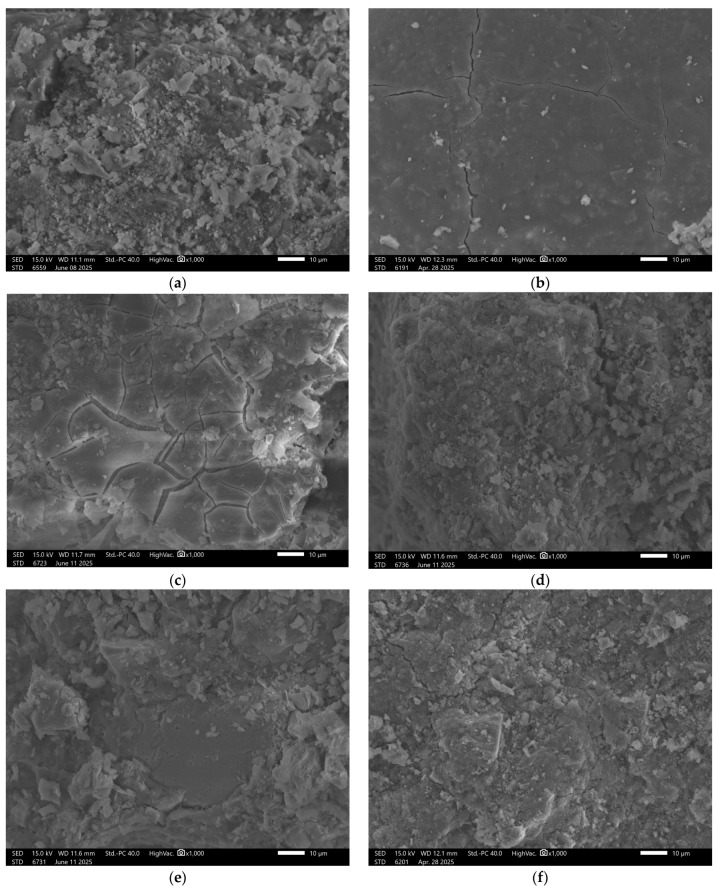
SEM Image for composition GST7: (**a**) reference sample; (**b**) H_2_O; (**c**) H_2_O + NaCl; (**d**) H_2_O + HCl; (**e**) H_2_O + HCl + CH_3_COOH + HNO_3_; (**f**) H_2_O + NaOH.

Sample GST 7, which contained a higher proportion of ground granulated blast furnace slag (GGBFS) than the previous samples, exhibited the best structural properties among the control samples, Figure 7.

Surface images show a smooth, dense texture without visible defects. Images taken after exposure to the previously mentioned solutions revealed some localized surface disintegration; however, the extent of this damage was lower than in samples with lower slag content.

**Figure 8:**
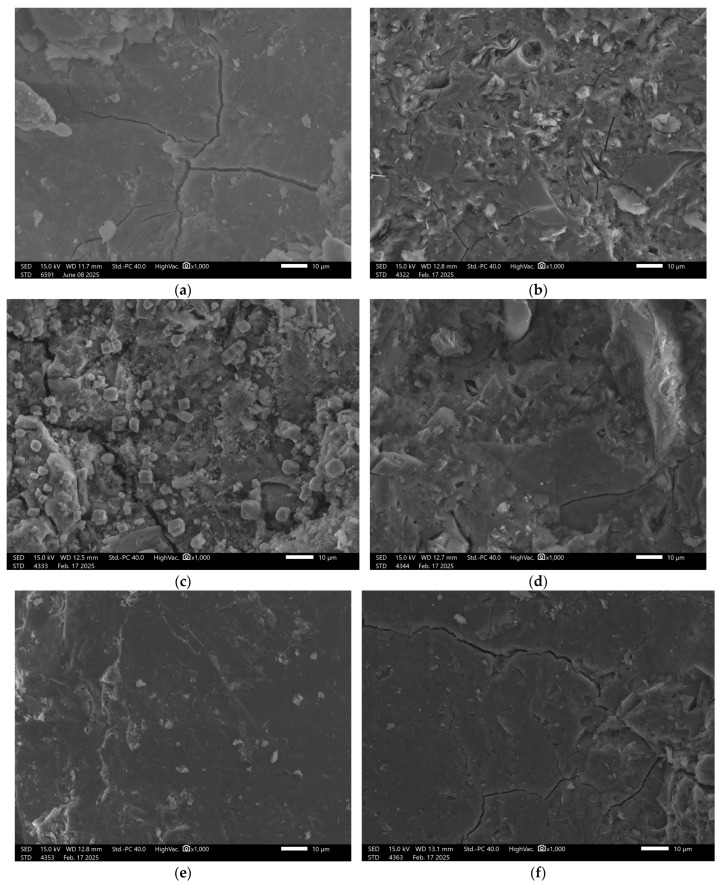
SEM Image for composition GEOV5: (**a**) reference sample; (**b**) H_2_O; (**c**) H_2_O + NaCl; (**d**) H_2_O + HCl; (**e**) H_2_O + HCl + CH_3_COOH + HNO_3_; (**f**) H_2_O + NaOH.

In contrast, analysis of GEO V5 samples, which did not contain slag, revealed a markedly different structure. The recorded surface showed significant porosity and a non-uniform matrix. In the reference state, numerous micropores and unreacted particles were visible, Figure 8.

Exposure to all test solutions led to ongoing degradation, with evident surface irregularities and signs of advanced deterioration clearly confirming the poor chemical durability of this mixture.

**Figure 9:**
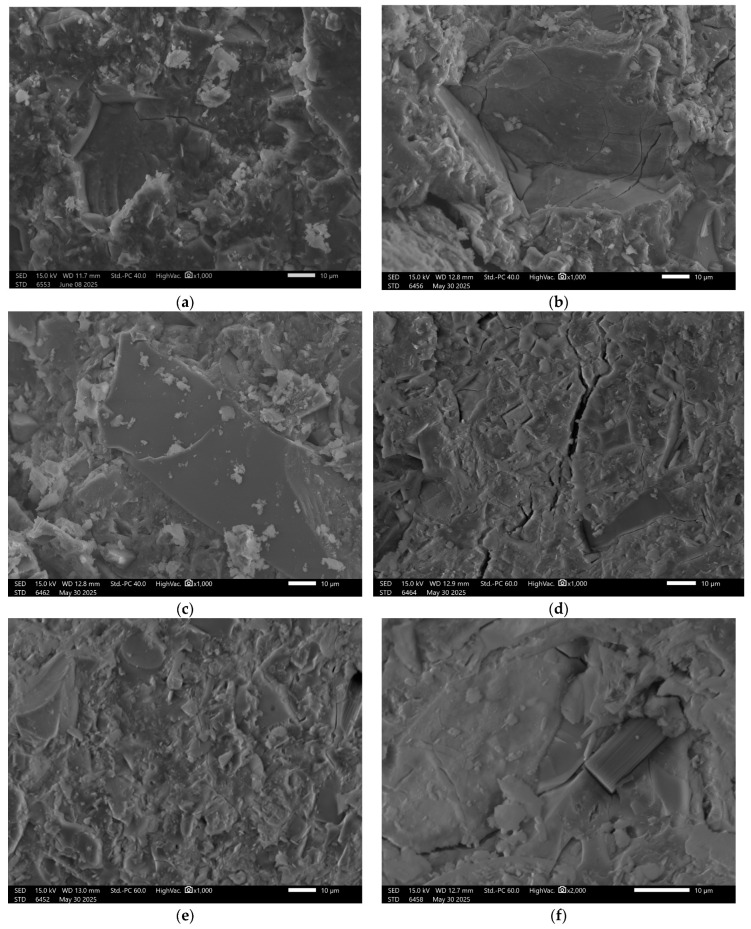
SEM Image for composition GEOV6: (**a**) reference sample; (**b**) H_2_O; (**c**) H_2_O + NaCl; (**d**) H_2_O + HCl; (**e**) H_2_O + HCl + CH_3_COOH + HNO_3_; (**f**) H_2_O + NaOH.

In the case of sample GEO V6, the structural composition was intentionally supplemented with blast furnace slag, Figure 9.

This modification resulted in a significant improvement in the surface quality of the reference sample, which appeared more compact and orderly. Although the solutions in which the remaining samples were immersed did lead to some degree of material degradation, a higher level of structural integrity was maintained compared to GEO V5.

In Figure 9f, there is visible carbon fiber. This addition was applied to all samples in a small amount to reinforce the mechanical properties. The good coherence fibers into the matrix is visible with minimal signs of interfacial voids or debonding. According to the literature, the main mechanism responsible for this phenomenon are Van der Waals forces, which facilitated close interfacial contact during curing, despite the absence of chemical bonding or surface functionalization [48,49]. Other confirmation for Van der Waals forces can be fiber imprints visible in the fractured surface morphology, Figure 6a. This imprint can be an effective enhancement to effective fiber-matrix adhesion enabled by van der Waals interactions, which contributed to improved load transfer and crack-bridging capability of the fibers. It also should be stressed that van der Waals forces are weak bonding, but in the case of fibers, they have a cumulative effect across the fiber surface area that can be substantial, especially when fiber dispersion and matrix wetting are well-controlled, as in the case of geopolymer composites investigated in a water environment [48,50].

**Figure 10:**
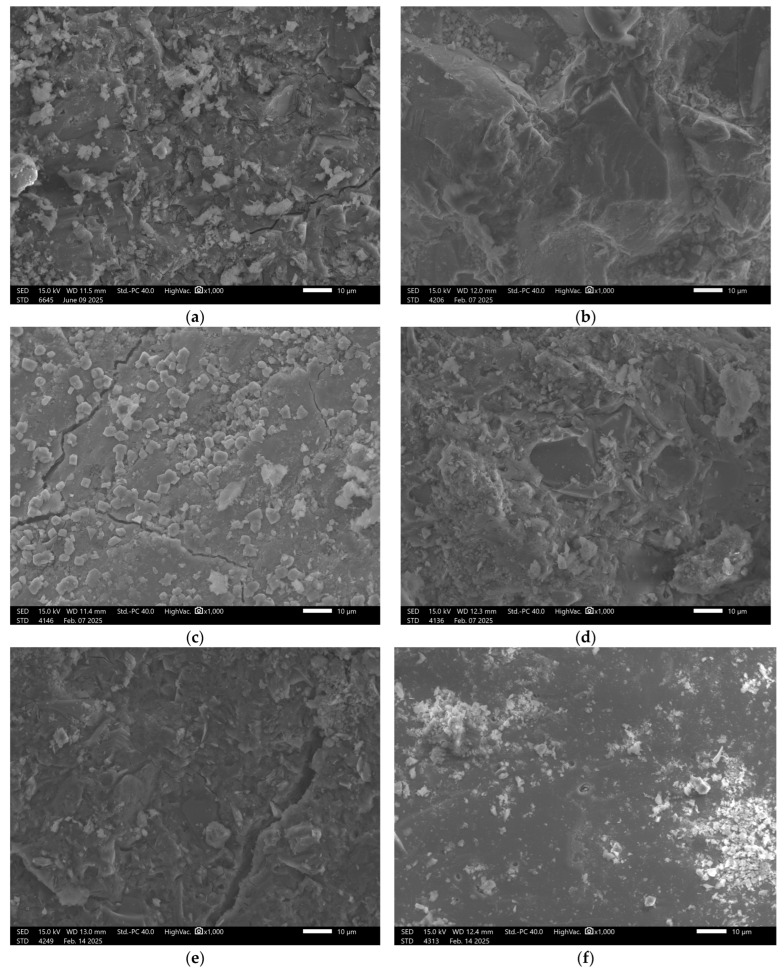
SEM Image for composition GEOV7: (**a**) reference sample; (**b**) H_2_O; (**c**) H_2_O + NaCl; (**d**) H_2_O + HCl; (**e**) H_2_O + HCl + CH_3_COOH + HNO_3_; (**f**) H_2_O + NaOH.

Similar results were obtained for sample GEO V7, Figure 10.

In the case of the material containing amphibolite, the observed surface exhibited a partially dispersed structure, where not all components had fully dissolved, resulting in visible agglomerates. After exposure to both acidic and alkaline solutions, significant surface damage was observed. Structural degradation occurred, with clearly defined areas showing more pronounced deterioration. This further emphasizes the importance of material composition in determining the chemical durability of geopolymer matrices and confirms the stabilizing role of slag in enhancing structural integrity.

The carbon fibers demonstrated good coherence with the matrix; however, their difficult identification in the SEM images may indicate uneven distribution. The potential agglomerations can weaken the material structure due to the uneven stress distribution, which consequences in lowering the mechanical properties of the material. This phenomenon very often appears in the case of the addition of the fibers to geopolymer composites [51]. This non-uniform dispersion could contribute to variability in the mechanical properties throughout the material’s volume.

It is obvious that an SEM figure cannot explain the chemical mechanisms (such as bond breaking or formation of new functional groups) responsible for the observed degradation and increased porosity. However, they are hopeful of the confirmation of the physical effects of these mechanisms in the material.

## 4. Discussion

Based on the conducted water absorption and compressive strength tests, it was possible to assess the suitability of the analyzed geopolymer composites for applications in aquatic environments, including both inland and marine waters [52,53].

It is worth mentioning that the mechanisms of degradation in various environments are slightly different for alkali, salts, and acids. In the case of acid conditions, the degradation mechanisms are connected with the breakdown of their aluminosilicate framework, particularly Si–O–Al bonds, which are susceptible to chemical attack in low-pH environments on leaching and hydrolysis phenomena [54,55]. The basic mechanism is connected with penetrating the geopolymer matrix by hydrogen ions (H^+^) and their exchange reactions with alkali cations such as Na^+^, K^+^, Ca^2+^ in the aluminosilicate network [54,55]. In this case, a confirmation of this phenomenon can be made by FTIR, where shift or reduction in Si–O–Al band intensity (950–1000 cm^−1^), indicating bond cleavage [56]. The reduction can be observed for HCl solution for the investigated compositions (Figure 3).

Other mechanisms are observed for alkaline solutions. In this case, the main reason responsible for the decrease in material properties is complex and includes degradation of Si–O–Si and Si–O–Al bonds under high concentrations of OH^−^ and the leaching of soluble silicates (especially leaching Na^+^ or K^+^). It caused gel disintegration under exposure to high-pH environments and, as a consequence, gradual dissolution of the aluminosilicate network [57,58]. Dome investigations suggest that the appearance in the geopolymer Ca systems can prevent this phenomenon and prevent the porosity increase and loss of mechanical strength [57,58]. The obtained results confirm that most of the mixtures with slag addition show better resistance in an alkali environment—especially the composition form GST group with slag addition (Figure 2).

Degradation in salts is dependent on particular saline environments and leads to various physical and chemical effects. The geopolymers are usually resistant to the mechanism that involves sodium salts, including marine environment salts (NaCl), which were investigated in the article. The material resistance is connected with high Na^+^ concentration in many geopolymers and their composites [58]. However, the provided research does not show this behavior for all types of samples. In the case of composition GST1 and GEOV5, the mechanical properties after exposure in a salt environment drop down. It can be caused by increasing the osmotic pressure of Na^+^ ions, physical distress, including cracking or efflorescence [58]. It should be noted that this phenomenon is not fully explained and would require in-depth research on the developed compositions.

The sample GEO V5 exhibited the lowest water absorption, suggesting a compact microstructure and limited porosity, and, thus, high resistance to the penetration of aqueous solutions. This is a significant parameter in the context of protecting structural elements exposed to wet–dry cycles and the migration of ions that can initiate corrosion processes, such as in reinforced steel.

Samples GST 1–7, particularly those with a lower slag content, showed increased water absorption, which may indicate reduced durability under long-term exposure to aqueous environments. The compressive strength results confirm that the blast furnace slag content plays a crucial role in the mechanical stability and chemical resistance of the material. The GST 1 series, which contained the highest amount of slag, exhibited the greatest strength, indicating effective bonding of reactive phases and the formation of a durable geopolymer matrix. Moreover, this series maintained good strength even after exposure to low-pH solutions, which is particularly important when designing structures intended for contact with acidic waters such as those around shipwrecks or bottom sediments contaminated with organic and inorganic compounds. In the case of sample GEO V6, enriching the base composition with slag improved its mechanical performance, clearly confirming the beneficial effect of this additive on the composite’s durability. This is a highly important observation in the context of underwater engineering, where the material must not only be resistant to water, but also maintain structural integrity under variable loads and erosive environmental conditions. Conversely, sample GEO V7, which contained amphibolite, demonstrated lower compressive strength despite the presence of slag. This may indicate a negative impact of this additive on the composite’s microstructure. It suggests that not all mineral fillers, which may enhance physical resistance in dry environments, perform well under submerged conditions. In this case, the amphibolite may have introduced undesirable microcracks or facilitated the penetration of aqueous solutions.

The provided research allows for characterization of a selected composition as a proper material for application in a water environment, thanks to a combination of properties such as a low water absorption and high mechanical strength. Additionally, the stability of these materials in chemically aggressive environments gives a possibility for application as a sewage infrastructure. While the material demonstrates promising performance under submerged laboratory conditions, successful field deployment at scale will require careful attention to material consistency, on-site handling, curing conditions, and regulatory acceptance. Before the application, additional research is required, including evaluating the long-term performance of these composites, accelerated aging tests to simulate multi-year service life. Also, on a wider scale, some problems with scaling up the solution can appear, including the following:

- The consistent and sustainable sourcing of raw materials, particularly the aluminosilicate precursors, on an industrial scale.
- Geopolymer formulations can be sensitive to mixing procedures, particularly due to their high alkalinity and relatively low water content. It could be hard to control in industrial conditions.
- Effective curing of geopolymers often requires controlled temperature and humidity conditions that are difficult to maintain on-site, especially in marine environments with fluctuating moisture, salinity, and wind exposure.
- Quality control processes that ensure consistent quality and performance across large volumes of material.
- Standardized procedures for testing geopolymers are still evolving, which can pose regulatory and certification challenges.

Further research should be focused on scaling up this solution and expanding the area of application. One of the possible directions is harbor infrastructure, in this case, also investigation of fire properties can be beneficial [59].

## 5. Conclusions

The geopolymer composites with a high content of blast furnace slag and without the addition of amphibolite GST 1 and GEO V6 proved to be the most suitable for underwater applications. They offer a combination of low water absorption, high mechanical strength, and stability in chemically aggressive environments. Moreover, the provided research allows us to formulate the following conclusions:

- Increasing the content of ground granulated blast furnace slag (GGBFS) in geopolymers enhances their compressive strength;
- The highest strength parameters were achieved with a slag content of 50%;
- The addition of amphibolite has a negative impact on compressive strength;
- The presence of slag and amphibolite additives leads to a slight increase in water absorption;
- FTIR analysis revealed characteristic spectra typical of geopolymer materials.

Based on the conducted research, it has been demonstrated that the analyzed materials can be used in various aquatic environments.

## Figures and Tables

**Table 1:** Sample designation.

No.	Sample	Metakaolin [kg]	Amphibolite [kg]	Sodium Water Glass [kg]	Silicon Dioxide [kg]	Carbon Fibers [kg]	Cellulose [kg]	Slag [kg]
1	GST 1	1	0	0.9	0.08	0.01	0.01	1
2	GST 3	1	0	0.9	0.08	0.01	0.01	0.3
3	GST 5	1	0	0.9	0.08	0.01	0.01	0.5
4	GST 7	1	0	0.9	0.08	0.01	0.01	0.7
5	Geo V5	1	0	0.9	0	0.01	0	0
6	Geo V6	1	0	0.9	0	0.01	0	1
7	Geo V7	1	1	0.9	0	0.01	0	0

**Table 2:** Density measurements.

Sample	Density [g/cm^3^]	Standard Deviation
GST1	1.952	0.014
GST3	1.756	0.036
GST5	1.627	0.022
GST7	1.807	0.026
GEOV5	1.867	0.018
GEOV6	1.900	0.021
GEOV7	1.826	0.030

**Table 3:** Oxide composition of the reference samples obtained via XRF analysis.

Oxide	GST 1	GST 3	GST 5	GST 7	GEO V5	GEO V6	GEO V7
MgO	1.95%	3.42%	3.02%	3.65%	0.18%	3.38%	0.00%
Al_2_O_3_	16.69%	19.56%	19.88%	20.27%	24.47%	21.31%	17.02%
SiO_2_	55.19%	47.34%	46.29%	45.06%	49.90%	40.87%	56.95%
P_2_O_5_	0.60%	0.025%	0.00%	0.11%	0.00%	0.19%	0.00%
SO_3_	0.20%	0.32%	0.24%	0.32%	0.34%	0.26%	0.22%
Cl	0.03%	0.03%	0.03%	0.03%	0.00%	0.02%	0.01%
K_2_O	4.58%	7.18%	6.73%	6.01%	7.36%	5.75%	6.52%
CaO	12.90%	16.50%	16.51%	16.22%	13.47%	17.21%	14.80%
Sc_2_O_3_	0.03%	0.05%	0.05%	0.05%	0.02%	0.04%	0.03%
TiO_2_	0.90%	1.10%	1.15%	1.13%	1.73%	1.27%	0.92%
V_2_O_5_	0.11%	0.08%	0.11%	0.12%	0.06%	0.16%	0.02%
Cr_2_O_3_	0.22%	0.07%	0.09%	0.11%	0.14%	0.15%	0.06%
MnO	0.21%	0.28%	0.29%	0.28%	0.22%	0.30%	0.25%
Fe_2_O_3_	4.82%	3.03%	4.18%	4.91%	1.81%	6.70%	3.04%
NiO	0.03%	0.01%	0.01%	0.01%	0.04%	0.01%	0.01%
CuO	0.04%	0.02%	0.03%	0.04%	0.00%	0.05%	0.00%
ZnO	0.01%	0.01%	0.01%	0.01%	0.01%	0.01%	0.01%
SrO	0.05%	0.05%	0.06%	0.06%	0.04%	0.08%	0.04%
Y_2_O_3_	0.00%	0.01%	0.01%	0.01%	0.01%	0.01%	0.01%
ZrO_2_	1.32%	0.81%	1.21%	1.47%	0.07%	2.06%	0.05%
MoO_3_	0.02%	0.01%	0.01%	0.01%	0.01%	0.02%	0.00%

**Table 4:** Initial mass, final mass, and water absorption of the samples.

Sample	Solution	Initial Mass [g]	Final Mass [g]	Change [%]
GST1	H_2_O	50.49	54.27	7.49
	H_2_O + NaCl	49.52	52.85	6.71
	H_2_O + HCl	50.48	53.93	6.84
	H_2_O + HCl + CH_3_COOH + HNO_3_	50.50	53.31	5.56
	H_2_O + NaOH	52.01	55.54	6.78
GST3	H_2_O	49.19	52.64	7.01
	H_2_O + NaCl	48.22	52.01	7.89
	H_2_O + HCl	49.16	52.06	5.90
	H_2_O + HCl + CH_3_COOH + HNO_3_	47.86	50.52	5.56
	H_2_O + NaOH	49.64	52.48	5.71
GST5	H_2_O	45.42	49.77	9.65
	H_2_O + NaCl	42.90	47.53	10.81
	H_2_O + HCl	42.99	47.97	11.62
	H_2_O + HCl + CH_3_COOH + HNO_3_	43.66	47.67	9.18
	H_2_O + NaOH	43.35	48.04	10.82
GST7	H_2_O	53.22	56.91	6.94
	H_2_O + NaCl	52.53	55.90	6.42
	H_2_O + HCl	53.06	56.02	5.57
	H_2_O + HCl + CH_3_COOH + HNO_3_	53.15	55.76	4.90
	H_2_O + NaOH	52.95	55.99	5.73
GEOV5	H_2_O	53.98	55.77	3.31
	H_2_O + NaCl	54.25	55.93	3.10
	H_2_O + HCl	54.69	56.33	3.01
	H_2_O + HCl + CH_3_COOH + HNO_3_	52.79	53.69	1.70
	H_2_O + NaOH	51.69	52.89	2.31
GEOV6	H_2_O	50.21	53.28	6.13
	H_2_O + NaCl	49.68	52.43	5.54
	H_2_O + HCl	50.77	53.55	5.46
	H_2_O + HCl + CH_3_COOH + HNO_3_	49.86	51.87	4.04
	H_2_O + NaOH	50.35	53.27	5.79
GEOV7	H_2_O	49.21	52.58	6.84
	H_2_O + NaCl	49.90	53.59	7.40
	H_2_O + HCl	49.63	53.19	7.16
	H_2_O + HCl + CH_3_COOH + HNO_3_	49.01	51.8	5.69
	H_2_O + NaOH	49.11	52.61	7.12

## Data Availability

The original contributions presented in this study are included in the article. Further inquiries can be directed to the corresponding author.

## Appendix A

### Appendix A.1. Additional Test Results

**Table A1:** Elemental composition of the reference samples obtained via XRF analysis.

Element	GST 1	GST 3	GST 5	GST 7	GEO V5	GEO V6	GEO V7
Mg	3.96%	6.34%	6.09%	7.05%	4.47%	7.03%	3.18%
Al	13.28%	14.97%	15.21%	15.66%	18.72%	16.04%	13.39%
Si	41.83%	34.72%	33.27%	32.60%	37.45%	28.74%	42.59%
P	0.00%	0.00%	0.00%	0.00%	0.00%	0.01%	0.00%
S	0.14%	0.25%	0.18%	0.25%	0.29%	0.20%	0.19%
Cl	0.08%	0.06%	0.07%	0.06%	0.02%	0.03%	0.03%
K	7.70%	11.20%	10.46%	9.18%	11.97%	8.52%	10.73%
Ca	19.65%	23.74%	23.41%	22.56%	20.41%	23.17%	22.52%
Sc	0.06%	0.08%	0.08%	0.07%	0.05%	0.07%	0.05%
Ti	1.23%	1.42%	1.46%	1.40%	2.34%	1.52%	1.26%
V	0.15%	0.10%	0.13%	0.15%	0.08%	0.19%	0.04%
Cr	0.36%	0.12%	0.14%	0.16%	0.23%	0.22%	0.10%
Mn	0.41%	0.50%	0.49%	0.47%	0.41%	0.49%	0.48%
Fe	8.10%	4.74%	6.45%	7.40%	3.00%	9.78%	5.09%
Ni	0.07%	0.02%	0.02%	0.02%	0.07%	0.03%	0.03%
Cu	0.07%	0.05%	0.06%	0.07%	0.00%	0.09%	0.00%
Zn	0.02%	0.01%	0.02%	0.02%	0.03%	0.02%	0.02%
Sr	0.10%	0.10%	0.11%	0.12%	0.08%	0.14%	0.08%
Y	0.01%	0.01%	0.02%	0.02%	0.01%	0.02%	0.02%
Zr	2.54%	1.40%	2.10%	2.48%	0.13%	3.38%	0.09%
Mo	0.03%	0.01%	0.00%	0.01%	0.02%	0.02%	0.01%

## References

[1] Holechek J.L., Geli H.M.E., Sawalhah M.N., Valdez R. (2022). A Global Assessment: Can Renewable Energy Replace Fossil Fuels by 2050?. Sustainability.

[2] Albayrak E., Özen S. (2025). Enhancing Geopolymer Synthesis through Calcination: Increasing the Potential of Natural Material Utilization. Open Ceram..

[3] Peng J., Chen W., Dong B., Wang Y. (2025). Alkali-Activated Sand Washing Slurry-Based Geopolymers: Mechanical Strength, Microstructure and Environmental Impact. Constr. Build. Mater..

[4] Shehata N., Mohamed O.A., Sayed E.T., Abdelkareem M.A., Olabi A.G. (2022). Geopolymer Concrete as Green Building Materials: Recent Applications, Sustainable Development and Circular Economy Potentials. Sci. Total Environ..

[5] Philip S., Nidhi M. (2023). A Review on the Material Performance of Geopolymer Concrete as Green Building Materials. Mater. Today Proc..

[6] Criado M., Provis J.L. (2018). Alkali Activated Slag Mortars Provide High Resistance to Chloride-Induced Corrosion of Steel. Front. Mater..

[7] Elfadaly E., Othman A.M., Aly M.H., Elgarhy W.A., Abdellatief M. (2025). Assessing Performance and Environmental Benefits of High-Performance Geopolymer Mortar Incorporating Pumice and Rice Straw Ash. Sustain. Chem. Pharm..

[8] Fan X., Zhu J., Gao X. (2024). Sea/Coral Sand in Marine Engineered Geopolymer Composites: Engineering, Mechanical, and Microstructure Properties. Int. J. Appl. Ceram. Tech..

[9] Liu C., Huang X., Wu Y.-Y., Deng X., Zheng Z. (2021). The Effect of Graphene Oxide on the Mechanical Properties, Impermeability and Corrosion Resistance of Cement Mortar Containing Mineral Admixtures. Constr. Build. Mater..

[10] Huang T., Sun Z. (2021). Advances in Multifunctional Graphene-Geopolymer Composites. Constr. Build. Mater..

[11] Arbi K., Nedeljković M., Zuo Y., Ye G. (2016). A Review on the Durability of Alkali-Activated Fly Ash/Slag Systems: Advances, Issues, and Perspectives. Ind. Eng. Chem. Res..

[12] Gevaudan J.P., Craun Z., Srubar W.V. (2021). Sulfuric Acid Degradation of Alkali-Activated Metakaolin Cements Supplemented with Brucite. Cem. Concr. Compos..

[13] Bakharev T. (2005). Durability of Geopolymer Materials in Sodium and Magnesium Sulfate Solutions. Cem. Concr. Res..

[14] Shamsah M., Kalfat R., Subramaniam K.V.L. (2025). Impact of Low NaOH Molarities on Mechanical and Durability Properties of Ambient and Oven-Cured Fly Ash Geopolymer Concrete. J. Build. Eng..

[15] Frieda F.S., Greeshma S. (2025). Effects on the Strength and Durability of Graphene Oxide Modified Geopolymer Concrete Using Industrial Waste Bauxite Tailings. Case Stud. Constr. Mater..

[16] Tay C.H., Mazlan N., Wayayok A., Basri M.S., Albakri Abdullah M.M. (2025). Zeolite Based Foamed Geopolymer Concrete Reinforced with Cellulose Nanofibril Prepared in Low Concentration Alkaline Solution: Porosity, Compressive Strength, and Water Permeability. J. Clean. Prod..

[17] Ismail I., Bernal S.A., Provis J.L., San Nicolas R., Brice D.G., Kilcullen A.R., Hamdan S., Van Deventer J.S.J. (2013). Influence of Fly Ash on the Water and Chloride Permeability of Alkali-Activated Slag Mortars and Concretes. Constr. Build. Mater..

[18] Bernal S.A. (2015). The Resistance of Alkali-Activated Cement-Based Binders to Carbonation. Handbook of Alkali-Activated Cements, Mortars and Concretes.

[19] Lamaa G., Duarte A.P.C., Silva R.V., De Brito J. (2023). Carbonation of Alkali-Activated Materials: A Review. Materials.

[20] Morandeau A., Thiéry M., Dangla P. (2014). Investigation of the Carbonation Mechanism of CH and C-S-H in Terms of Kinetics, Microstructure Changes and Moisture Properties. Cem. Concr. Res..

[21] Bernal S.A., Provis J.L., Mejía De Gutiérrez R., Van Deventer J.S.J. (2015). Accelerated Carbonation Testing of Alkali-Activated Slag/Metakaolin Blended Concretes: Effect of Exposure Conditions. Mater. Struct..

[22] Albitar M., Mohamed Ali M.S., Visintin P., Drechsler M. (2017). Durability Evaluation of Geopolymer and Conventional Concretes. Constr. Build. Mater..

[23] Luga E., Mustafaraj E., Corradi M., Atiș C.D. (2024). Alkali-Activated Binders as Sustainable Alternatives to Portland Cement and Their Resistance to Saline Water. Materials.

[24] Amran M., Al-Fakih A., Chu S.H., Fediuk R., Haruna S., Azevedo A., Vatin N. (2021). Long-Term Durability Properties of Geopolymer Concrete: An in-Depth Review. Case Stud. Constr. Mater..

[25] Zhang Y.H., Zhong W.L., Fan L.F. (2024). Long-Term Durability Investigation of Basalt Fiber-Reinforced Geopolymer Concrete in Marine Environment. J. Mater. Res. Technol..

[26] Korniejenko K., Halyag N., Mucsi G. (2019). Fly Ash as a Raw Material for Geopolymerisation-Chemical Composition and Physical Properties. IOP Conf. Ser. Mater. Sci. Eng..

[27] Burduhos Nergis D.D., Abdullah M.M.A.B., Vizureanu P., Tahir M.F.M. (2018). Geopolymers and Their Uses: Review. IOP Conf. Ser. Mater. Sci. Eng..

[28] Fang G., Ho W.K., Tu W., Zhang M. (2018). Workability and Mechanical Properties of Alkali-Activated Fly Ash-Slag Concrete Cured at Ambient Temperature. Constr. Build. Mater..

[29] Indhumathi Anbarasan M., Leema Margret A., Ragavan V., Ramprashath J. (2023). Investigation on Corrosion Behaviour of Geopolymer Concrete Using DMS and M−Sand as a Fine Aggregate under Ambient Curing Conditions. Mater. Today Proc..

[30] Zhang B., Yu T., Guo H., Chen J., Liu Y., Yuan P. (2022). Effect of the SiO_2_/Al_2_O_3_ Molar Ratio on the Microstructure and Properties of Clay-Based Geopolymers: A Comparative Study of Kaolinite-Based and Halloysite-Based Geopolymers. Clays Clay Miner..

[31] Khan R., Iqbal S., Soliyeva M., Ali A., Elboughdiri N. (2025). Advanced Clay-Based Geopolymer: Influence of Structural and Material Parameters on Its Performance and Applications. RSC Adv..

[32] Setlak K., Mikuła J., Łach M. (2023). Application of Industrial Waste Materials by Alkaline Activation for Use as Geopolymer Binders. Materials.

[33] Al-Noaimat Y.A., Ghaffar S.H., Chougan M., Al-Kheetan M.J. (2023). A Review of 3D Printing Low-Carbon Concrete with One-Part Geopolymer: Engineering, Environmental and Economic Feasibility. Case Stud. Constr. Mater..

[34] Asadizadeh M., Hedayat A., Tunstall L., Gonzalez J.A.V., Alvarado J.W.V., Neira M.T. (2024). The Impact of Slag on the Process of Geopolymerization and the Mechanical Performance of Mine-Tailings-Based Alkali-Activated Lightweight Aggregates. Constr. Build. Mater..

[35] Mohamed O., Ahmed E., Najm O., Al-Aribe K., Hijah E. (2023). Water Absorption Characteristics and Rate of Strength Development of Mortar with Slag-Based Alkali-Activated Binder and 25% Fly Ash Replacement. Mater. Today Proc..

[36] Gastoł W., Shalomieiev V.A., Tabunschyk G.V., Łach M., Kozub B., Nykiel M., Korniejenko K. (2025). Evaluation of the Possibility of Preparing Geopolymer Materials Based on Slags and Fly Ashes from the Thermal Treatment of Municipal Waste. Mater. Werkst..

[37] Liu J., Lv C. (2022). Durability of Cellulosic-Fiber-Reinforced Geopolymers: A Review. Molecules.

[38] Mu S., Liu J., Liu J., Wang Y., Shi L., Jiang Q. (2018). Property and Microstructure of Waterborne Self-Setting Geopolymer Coating: Optimization Effect of SiO2/Na2O Molar Ratio. Minerals.

[39] Korniejenko K., Kejzlar P., Louda P. (2022). The Influence of the Material Structure on the Mechanical Properties of Geopolymer Composites Reinforced with Short Fibers Obtained with Additive Technologies. Int. J. Mol. Sci..

[40] Wang X., He X., Wang X. (2023). FTIR Analysis of the Functional Group Composition of Coal Tar Residue Extracts and Extractive Residues. Appl. Sci..

[41] Bredács M., Barretta C., Castillon L.F., Frank A., Oreski G., Pinter G., Gergely S. (2021). Prediction of Polyethylene Density from FTIR and Raman Spectroscopy Using Multivariate Data Analysis. Polym. Test..

[42] Yusuf M.O. (2023). Bond Characterization in Cementitious Material Binders Using Fourier-Transform Infrared Spectroscopy. Appl. Sci..

[43] Thomsen R.M., Skibsted J., Yue Y. (2018). The Charge-Balancing Role of Calcium and Alkali Ions in Per-Alkaline Aluminosilicate Glasses. J. Phys. Chem. B.

[44] Toniolo N., Rincón A., Roether J.A., Ercole P., Bernardo E., Boccaccini A.R. (2018). Extensive Reuse of Soda-Lime Waste Glass in Fly Ash-Based Geopolymers. Constr. Build. Mater..

[45] Onutai S., Osugi T., Sone T. (2023). Alumino-Silicate Structural Formation during Alkali-Activation of Metakaolin: In-Situ and Ex-Situ ATR-FTIR Studies. Materials.

[46] Finocchiaro C., Barone G., Mazzoleni P., Leonelli C., Gharzouni A., Rossignol S. (2020). FT-IR Study of Early Stages of Alkali Activated Materials Based on Pyroclastic Deposits (Mt. Etna, Sicily, Italy) Using Two Different Alkaline Solutions. Constr. Build. Mater..

[47] Amar M., Ladduri B., Alloul A., Benzerzour M., Abriak N.-E. (2024). Microstructure and Mechanical Properties of Geopolymers Utilizing Excavated Soils, Metakaolin and Slags. J. Build. Eng..

[48] Guan X., Wu J.-Q., Hernandez A.G., Li B., Do H. (2022). Molecular Dynamic Simulations of Interfacial Interaction Mechanism between the NASH Gels and the Polyethene Fibre. Constr. Build. Mater..

[49] Kai M.F., Zhang L.W., Liew K.M. (2020). Carbon Nanotube-Geopolymer Nanocomposites: A Molecular Dynamics Study of the Influence of Interfacial Chemical Bonding upon the Structural and Mechanical Properties. Carbon.

[50] Sekkal W., Zaoui A. (2024). Residual Water and Interfacial Bonding Effects on the Mechanical Performance of CNT/Fly Ash Geopolymer Binder. Struct. Concr..

[51] Růžek V., Dostayeva A.M., Walter J., Grab T., Korniejenko K. (2023). Carbon Fiber-Reinforced Geopolymer Composites: A Review. Fibers.

[52] Rahman S.K., Al-Ameri R. (2023). Long-Term Performance of Basalt Fibre-Reinforced Marine Geopolymer Concrete in Harsh Environment. Mag. Concr. Res..

[53] Wardhono A., Risdianto Y., Sabariman B., Hidajati N.W., Andajani N. (2023). The Effect of Sodium Silicate to NaOH Ratio on Strength Development of Fly Ash Geopolymer Mortar in Marine Environment. E3S Web Conf..

[54] Aiken T.A., Gu L., Kwasny J., Huseien G.F., McPolin D., Sha W. (2022). Acid Resistance of Alkali-Activated Binders: A Review of Performance, Mechanisms of Deterioration and Testing Procedures. Constr. Build. Mater..

[55] Ariyadasa P.W., Manalo A.C., Lokuge W., Aravienthan V., Gerdes A., Kaltenbach J. (2025). Degradation Mechanisms of Low-Calcium Fly Ash-Based Geopolymer Mortar in Simulated Aggressive Sewer Conditions. Cem. Concr. Res..

[56] Ma Y.-K., Rigolet S., Michelin L., Paillaud J.-L., Mintova S., Khoerunnisa F., Daou T.J., Ng E.-P. (2021). Facile and Fast Determination of Si/Al Ratio of Zeolites Using FTIR Spectroscopy Technique. Microporous Mesoporous Mater..

[57] Provis J.L., Bernal S.A. (2014). Geopolymers and Related Alkali-Activated Materials. Annu. Rev. Mater. Res..

[58] Zhang B. (2024). Durability of Low-Carbon Geopolymer Concrete: A Critical Review. Sustain. Mater. Technol..

[59] Bolina F.L., Henn A.S. (2025). Eco-Friendly Reinforced Concrete Beams Exposed to Standardized Fire: A Thermal Finite Element Analysis. Sustainability.

